# What impact does postgraduate clinical training have on empathy among Japanese trainee dentists?

**DOI:** 10.1186/s12909-020-02481-y

**Published:** 2021-01-14

**Authors:** Toshiko Yoshida, Sho Watanabe, Takayuki Kono, Hiroaki Taketa, Noriko Shiotsu, Hajime Shirai, Yukie Nakai, Yasuhiro Torii

**Affiliations:** 1 Center for Education in Medicine and Health Sciences (Dental Education), Graduate School of Medicine, Dentistry, and Pharmaceutical Sciences, Okayama University, 2-5-1, Shikata-cho, Kita- ku, 700-8558 Okayama, Okayama, Japan; 2 Comprehensive Dental Clinic, Okayama University Hospital, 2-5-1, Shikata-cho, Kita-ku, 700-8558 Okayama, Okayama, Japan; 3 Department of Dental Hygiene, University of Shizuoka, Junior College, 2-2-1 Oshika, Suruga- ku, 422-8021 Shizuoka, Shizuoka, Japan

**Keywords:** Empathy, Trainee dentists, Clinical training, Jefferson Scale of Empathy, Roter interaction analysis system, Simulated patients

## Abstract

**Background:**

Enhancing empathy in healthcare education is a critical component in the development of a relationship between healthcare professionals and patients that would ensure better patient care; improved patient satisfaction, adherence to treatment, patients’ medication self-efficacy, improved treatment outcomes, and reduced patient anxiety. Unfortunately, however, the decline of empathy among students has been frequently reported. It is especially common when the curriculum transitions to a clinical setting. However, some studies have questioned the significance and frequency of this decline. Thus, the purpose of this study was to determine the impact of postgraduate clinical training on dental trainees’ empathy from cognitive, behavioral, and patients’ perspective.

**Methods:**

This study included 64 trainee dentists at Okayama University Hospital and 13 simulated patients (SPs). The trainee dentists carried out initial medical interviews with SPs twice, at the beginning and the end of their clinical training. The trainees completed the Japanese version of the Jefferson Scale of Empathy for health professionals just before each medical interview. The SPs evaluated the trainees’ communication using an assessment questionnaire immediately after the medical interviews. The videotaped dialogue from the medical interviews was analyzed using the Roter Interaction Analysis System.

**Results:**

No significant difference was found in the self-reported empathy score of trainees at the beginning and the end of the clinical training (107.73 [range, 85–134] vs. 108.34 [range, 69–138]; *p* = 0.643). Considering the results according to gender, male scored 104.06 (range, 88–118) vs. 101.06 (range, 71–122; *p* = 0.283) and female 109.17 (range, 85–134) vs. 111.20 (range, 69–138; *p* = 0.170). Similarly, there was no difference in the SPs’ evaluation of trainees’ communication (10.73 vs. 10.38, *p* = 0.434). Communication behavior in the emotional responsiveness category for trainees in the beginning was significantly higher than that at the end (2.47 vs. 1.14, *p* = 0.000).

**Conclusions:**

Overall, a one-year postgraduate dental training program neither reduced nor increased trainee dentists’ empathy levels. Providing regular education support in this area may help trainees foster their empathy.

## Background

Empathy is important in the relationship between healthcare professionals and patients, and is widely acknowledged as an efficient component of effective communication. Communicating with empathy helps the development of a therapeutic relationship. When patients are interviewed with empathic communication, they feel understood and accepted, and their concerns and problems are well elicited. If the healthcare professional understands the patient’s problems and how they view them, the professionals become more capable of making an accurate diagnosis, and formulating appropriate treatment plans [1, 2]. Empathy and its impact on professional communication are associated with improved patient satisfaction [3–5], adherence to treatment [6], patients’ medication self-efficacy [7], improved treatment outcomes [8], and reduced patient anxiety [9, 10].

Empathy is a multifaceted concept, which has cognitive, affective, and behavioral dimensions that were developed and integrated over time [11]. The Cambridge dictionary defines empathy as “the ability to share someone else’s feelings or experiences by imagining what it would be like to be in that person’s situation” [12]. However, its definition has not yet achieved consensus in the field.

Enhancing empathy is a critical concern in healthcare. Empathy can be fostered by raising awareness of the patient’s thoughts about the cause and importance of the patient’s problems. It can also be strengthened by acquiring appropriate listening skills to elicit patient’s concerns and problems. These skills help professionals treat patients with respect and in a caring manner which further leads to cultivation of humanistic behavior, one of the attributes of professionalism [13].

However, the decline of empathy levels among students during medical and dental education, especially after increased patient contact during clinical training, has frequently been demonstrated [14, 15]. Some factors contributing to the decline of empathy could be time constraints, patient care interactions, and a heavy load of study and work [16]. Yet, some reviews have suggested that the decline in empathy is considerably exaggerated. Díaz-Narváez and colleagues [17] reported various patterns of change in empathy levels during dental education. Colliver and colleagues [18] concluded that empathy decline may not be severe enough to affect patient care. To clarify this issue, we aimed to explore the impact of postgraduate clinical training that includes the opportunity to treat patients, on empathy among trainee dentists.

Most previous studies have used a single measurement for empathy, especially self-reported measures [16, 17, 19–25]. This may provide a limited understanding of empathy, because it is a multidimensional attribute. Colliver et al. [18] noted that the patients’ perceptions should be considered in assessing healthprofessionals’ empathy. Therefore, this study used three measures to assess empathy: the cognitive aspect, behavioral aspect, and patient perspective. The behavioral dimension was measured by the trainee dentists’ empathic communication, and the patients’ perspective was measured by the simulated patients’ (SPs) assessment of trainee communication during initial medical interviews.

The purpose of this study was to determine the impact of postgraduate clinical training on dental trainees’ empathy not just from a cognitive and behavioral perspective, but also from the patients’ perspective. To the best of our knowledge, this is the first study to examine changes in empathy during postgraduate training in dental education, assessed with multi-perspective measurements.

## Methods

### Participants

We chose the convenience sampling method for this study. The total number of trainee dentists that enrolled at the Okayama University Hospital for the one-year postgraduate clinical training course in 2017 and 2018 was 64. All 64 of them (18 males and 46 females) were recruited for the study. In addition, 13 SPs from the Okayama Working Group for Simulated Patients (11 females and two males) participated in the study. Ten SPs each participated in this study in 2017 and 2018, including seven SPs who participated both years. Participants were given both verbal and written explanations of the study and its procedure. All trainees provided their signed informed consent after confirming that they understand. All SPs provided consent via e-mail. Participants were reassured that choosing not to take part in the study would not affect their clinical training.

### Overview of the postgraduate clinical training course for dentists at Okayama University Hospital

After graduating from high school, dental students in Japan enroll in a six-year undergraduate program, followed by a mandatory one-year clinical training program after acquiring their license. This training is comprehensive and intended to equip the trainees with skills to provide general dental care for the entire oral cavity and an emphasis on patient-centered holistic care.

The postgraduate program consisted of a combination of departments that provide proficiency training in basic and common treatments encountered in daily practice. Under the supervision of the senior dentists, the trainee dentists assist with treatment and also treat patients directly. Completing a minimum number of cases for general dentistry basic practices is required. An electronic portfolio is used to encourage the trainees to review their practice critically. After each session, the trainees write the details of the treatment they performed, what they noticed in this practice, and what they should change to improve moving forward. The supervising senior dentists comment on their portfolios, adopting a reflective and supportive approach to facilitate trainee learning. Case presentations and instructive seminars are also required.

### Data collection procedure

Each trainee dentist conducted an initial medical interview with an SP twice: at the start and end of their training program. The medical interviews took place in a room in a medical office which is different from a consultation room. These medical interviews were a mandatory requirement of the regular training and were not specifically conducted for the purpose of research. However, the trainees consented to participating in this study and using the data from these interviews for this research.

Just before the initial medical interview with SPs, the trainee dentists completed the Japanese version of the Jefferson Scale of Empathy (JSE) for health professionals (HP-Version). Different dental problems were selected for presentation at the initial interview at the beginning of the training and the one at the end of the training. The former primarily presented concerns about the potential severity of persistent stomatitis on their tongues, while the latter primarily focused on the potential severity of persistent swelling and dull pain in their cheeks. We acknowledge that we should have examined the impact of the two different cases to further improve the validity of the study. The medical interviews were videotaped and had no time limitation. SPs evaluated the trainees’ communication using an assessment questionnaire immediately after the medical interviews.

### Measures

#### JSE (HP-Version): self‐assessment of trainees’ empathy

The JSE (HP-Version) is a self-reporting instrument developed to measure empathy specifically in physicians and health professionals [26]. Ample evidence has supported the reliability and validity of the JSE for students and healthcare professionals [27]. The JSE is an extensively used instrument that has been translated into 43 languages and used in over 60 countries [28]. The psychometric properties of its Japanese version have also been reported [29]. The internal consistency of the JSE for this study’s population was good; Cronbach’s alpha at the beginning and end of the training were 0.78 and 0.86, respectively.

The JSE consists of 20 items, each rated on a seven-point Likert-type scale (1 = *strongly disagree*, 7 = *strongly agree*), with possible total scores ranging from 20 to 140. Half of the items are reverse scored, so that an overall higher score shows a more empathic orientation toward patient care.

#### The Roter interaction analysis system (RIAS)

The RIAS was used to analyze the videotaped dialogue from the medical interviews. The RIAS is a method for coding medical dialogue and is most widely used in Western countries [30]. However, its applicability has also been reported for the Japanese population [31].

The dialogue is divided into ‘utterances’ that are defined as the smallest units in the medical interview. Units vary in length from single words to long sentences composed of one thought or piece of information. Each utterance falls into one of 41 mutually-exclusive code categories, excluding unintelligible utterances, according to the Japanese version of the RIAS [30]. In this study, six new categories were added to distinguish dental conversations from other medical conversations. The six new categories were originally included in the medical conversation, under both ‘Gathering medical data,’ and ‘Giving medical information.’ We then consolidated all categories into 14 larger composite clusters based on content similarity (Table 1).

**Table 1:** RIAS categories in this study

Cluster	Each category
Relationship building	Personal remarks, Social conversation, Remediation, Partnership statements, Self-disclosure statement
Positive talk	Laughing, Telling jokes, Showing direct approval, Providing general compliments, Showing agreement or understanding, Providing back-channel responses
Negative talk	Showing direct disapproval, Providing general criticisms
Emotional expression	Empathizing statements, Legitimizing statements, Showing concern or worry, Reassuring, Encouraging or showing optimism, Asking for reassurance
Facilitative behaviors	Providing orientation, Instructing, Paraphrasing/checking for understanding, Clarifying for understanding, Requesting repetition, Asking for opinions, Asking for permission, Using transition words, Requesting services or medication
Counseling/direction	Counseling or providing direction about any topic
Gathering medical data	Open or closed questions regarding medical conditions or therapeutic regimen
Gathering psychosocial data	Open or closed questions regarding psychosocial or lifestyle issues
Gathering dental data	Open or closed questions regarding current dental history ^a^ or past dental history ^a^
Gathering data gathering about other issues	Open or closed questions about other issues
Giving medical information	Providing information about medical conditions or therapeutic regimen
Giving psychosocial information	Providing information giving about psychosocial or lifestyle issues
Giving dental information	Providing information about current dental history ^a^ or past dental history ^a^
Giving information about other issues	Providing information about other issues

^a^ New category

Coding was performed directly from videotapes rather than transcripts; therefore, utterances can be categorized based on voice tone and phrasing cues as well as literal meaning.

Two coders (SW and TY) independently analyzed 20 videotapes that were not included in this study to assess inter-coder reliability. SW is a dentist with a PhD degree and TY is a faculty specialized in behavioral dentistry with a PhD degree and has a dental technician license. Both coders completed the RIAS coding training provided by RIAS Japan. Inter-class correlation coefficients were calculated between the results of the two coders for the categories with a mean frequency greater than two per medical interview. The average correlation was 0.69 (0.25–0.99) for trainee dentists and 0.74 (0.64–0.82) for SPs, indicating moderate coding reliability. In addition, 20 randomly selected videotapes in this study were independently double coded by the second coder (TY). The average correlation was 0.70 (0.46–0.84) for trainee dentists and 0.77 (0.49–0.88) for SPs, indicating moderate coding reliability. The main coder (SW) analyzed all videotapes in this study according to the RIAS Japan manual.

The frequency (the absolute number) of utterances for each category was used for the comparison instead of the percentage rate because there was no significant difference in the duration of the medical interview between the beginning and end of training.

#### SP assessment questionnaire of trainee dentists’ communication

The SP assessment questionnaire comprises five items included in Table 2, answered on a four-point scale (0 = *disagree*, 1 = *somewhat disagree*, 2 = *somewhat agree*, 3 = *agree*). The possible total scores ranged from 0 to 15, where a high score indicates a more positive assessment.

**Table 2:** Mean item score of SP Assessment

	Item	At the beginning		At the end		Wilcoxon	
		Mean	SD	Mean	SD	Z	P
1	Listens carefully while you are talking	2.13	0.75	2.19	0.69	-0.508	0.612
2	Understands your worries and uneasiness	2.03	0.67	1.70	0.75	-2.427	0.015**
3	Speaks with appropriate words and speed in plain language	2.23	0.64	2.25	0.62	-0.141	0.888
4	Treats you as an equal; never ‘talks down’ to you or treats you like a child	2.30	0.55	2.20	0.60	-0.974	0.330
5	Overall, would you see this dental trainee again?	2.05	0.65	2.03	0.73	-0.267	0.790
	Total	10.73	2.49	10.38	2.79	-0.782	0.434

**P < 0.01

This questionnaire was prepared based on the American Board of Internal Medicine’s Patient Assessment survey questionnaire, which consists of 10 items [32]. Since that questionnaire was not prepared exclusively for medical interviews, the five question items which matched the initial interview were selected and the language was modified to make it easier for the Japanese SPs to understand. Cronbach’s alpha at the beginning and the end of the training were 0.82 and 0.88, respectively, which indicated good internal consistency.

### Statistical analyses

The mean medical interview duration, the mean total JSE score, frequency of trainees’ and SPs’ utterances for each category, and total SP assessment score for the start and end of training were compared.

Paired t-test was used to evaluate the mean total JSE score because the data were normally distributed. The mean medical interview duration, the mean frequency of trainees’ and SPs’ utterances, and the mean SP assessment score were not expected to be normally distributed, and so the Wilcoxon signed-ranks test was utilized. All statistical analyses were conducted using the software SPSS version 24 (IBM, Tokyo, Japan). A significant difference was defined as > 0.05.

## Results

All 64 trainee dentists who were enrolled for the one-year postgraduate clinical training course at the Okayama University Hospital in 2017 and 2018 were recruited, and all of them (aged 24–39 years, 18 males and 46 females) were participated.

### Duration of the medical interview

The mean medical interview duration at the beginning and end of training were 8 minutes 44 seconds (SD, 2 minutes 25 seconds; range, 4 minutes 47 seconds to 16 minutes 35 seconds) and 8 minutes 7 seconds (SD, 2 minutes 27 seconds; range, 3 minutes 52 seconds to 15 minutes 41 seconds), respectively; these duration did not differ significantly (Z=-1.819; *p* = 0.069).

According to an existing study performed at a different dental school, the average duration for trainees was about 6 minutes [33], which is shorter than the times of our study. This may be due to the trainees in our study taking time to conduct the medical interview thoroughly.

### JSE

The mean JSE total score for all trainee participants, as well as by gender, is provided in Table 3. The JSE total score averaged 107.73 (SD, 10.59; range, 85–134) at the beginning and 108.34 (SD, 14.05; range, 69–138) at the end; no significant difference between the two administrations is observed. Considering gender, there were also no significant differences between the two timepoints among male (104.06 [range, 88–118] vs. 101.06 [range, 71–122]) or among female (109.17[ range, 85–134] vs. 111.20 [range 69–138]).

**Table 3:** Mean JSE total score for all participants and by gender

	At the beginning		At the end		t-test	
	Mean	*SD*	Mean	*SD*	t	P
All subjects (*n* = 64)	107.73	10.59	108.34	14.05	-0.466	0.643
Male (*n* = 18)	104.06	8.21	101.06	14.55	1.109	0.283
Female (*n* = 46)	109.17	11.13	111.20	12.91	-1.396	0.170

### RIAS

Tables 4 and 5 show the mean frequencies of trainees’ and SPs’ utterances for the clusters, respectively. The cluster names are shown in quotation marks in this article. There were no differences in the total number of trainees’ and SPs’ utterances between the two timepoints.

**Table 4:** Mean frequencies of clusters of RIAS categories for trainee dentists at the start and end of training

Clusters	At the start		At the end		Wilcoxon	
	Mean	*SD*	Mean	*SD*	Z	P
Total utterances	94.44	22.78	93.50	28.76	-0.792	0.428
Relationship building	4.98	1.78	4.84	1.92	-0.662	0.508
Positive talk	37.20	14.02	36.31	15.47	-0.694	0.487
Negative talk	0.00	0.00	0.02	0.13	-1.000	0.317
Emotional expression	2.47	2.17	1.14	1.53	-3.948	0.000**
Facilitative behaviors	21.55	7.24	21.08	8.74	-0.630	0.528
Counseling/direction	0.00	0.00	0.00	0.00	0.000	1.000
Gathering medical data	6.59	2.93	5.19	2.41	-2.990	0.003**
Gathering psychosocial data	1.23	1.39	0.75	0.99	-2.544	0.011*
Gathering dental data	20.00	5.43	23.52	6.94	-3.164	0.002**
Gathering data about other issues	0.00	0.00	0.00	0.00	0.000	1.000
Providing medical information	0.13	0.42	0.20	0.62	-0.659	0.510
Providing psychosocial information	0.02	0.13	0.05	0.28	-0.816	0.414
Providing dental information	0.27	0.67	0.39	1.16	-0.876	0.381
Providing information about other issues	0.00	0.00	0.02	0.13	-1.000	0.317

*P < 0.05, **P < 0.01

**Table 5:** Mean frequencies of clusters of RIAS categories for SPs at the start and end of the training

Clusters	At the start		At the end		Wilcoxon	
	Mean	*SD*	Mean	*SD*	Z	P
Total utterances	79.36	20.27	79.34	23.73	-0.182	0.855
Relationship building	2.39	1.05	2.25	1.05	-0.821	0.412
Positive talk	27.34	11.57	26.59	12.56	-0.041	0.967
Negative talk	0.45	0.78	0.61	0.92	-1.127	0.260
Emotional expression	1.23	1.34	0.63	0.88	-2.877	0.004**
Facilitative behaviors	1.19	1.15	1.00	0.99	-1.136	0.256
Counseling/direction ^a^	-	-	-	-	-	-
Gathering medical data	0.08	0.32	0.00	0.00	-1.890	0.059
Gathering psychosocial data	0.00	0.00	0.00	0.00	0.000	1.000
Gathering dental data	0.09	0.43	0.11	0.36	-0.707	0.480
Gathering data about other issues	0.00	0.00	0.00	0.00	0.000	1.000
Providing medical information	8.64	3.71	6.08	2.60	-4.095	0.000**
Providing psychosocial information	5.70	3.70	2.75	2.08	-4.799	0.000**
Providing dental information	32.23	8.66	39.33	11.02	-3.806	0.000**
Providing information about other issues	0.00	0.00	0.00	0.00	0.000	1.000

**P < 0.01

^a^ Category for dentists only

Compared with the trainee dentists at the start of their training, those at the end had less ‘Emotional expression’ by half (2.47 vs. 1.14), which included empathic and legitimizing statements. They were also less involved in ‘Gathering medical data’ pertinent medical conditions or therapeutic regimen issues as suggested by the drop in the score from 6.59 to 5.19 and ‘Gathering psychosocial data’ regarding psychosocial or lifestyle issues as suggested by the drop in the score from 1.23 to 0.75. However, they engaged in more ‘Gathering dental data’ including current or past dental history as indicated by the increase in the score from 20.00 to 23.52.

Consistent with the trainees’ results, SPs provided fewer ‘Emotional expression’ statements nearly by half (scores dropped from 1.23 to 0.63), including expressing their concerns and less ‘Providing medical information’ (scores dropped from 8.64 to 6.08) and ‘Providing psychosocial information’ by half (scores dropped from 5.70 to 2.75). However, they provided more dental data in the medical interview at the end of training (32.23 vs. 39.33).

### SP assessment of trainee dentists’ communication

The individual item scores of the SP assessment at the beginning and the end of the training are shown in Table 2, which shows that the mean total scores of SP assessment at the beginning and end of the training were 10.73 (*SD*, 2.49; range, 6–15) and 10.38 (*SD*, 2.79; range, 5–15), respectively. No significant difference in mean total score was found between the two administrations. Only the score for the item ‘Did you feel your worries and anxiety were understood?’ was significantly lower at the end of training compared to the beginning.

## Discussion

We examined what impact, as assessed by three indicators, does the course of a one-year postgraduate clinical training program have on empathy among Japanese trainee dentists. This study found that trainees’ self-reported empathy levels remained static, and communication behavior decreased in the emotional responsiveness category during trainees’ medical interviews. Additionally, the total score of SP assessment of trainees’ communication remained unchanged; however, there was a decline in trainees’ attitudes about accepting SPs’ concerns and anxiety from the SPs’ perspectives.

Although many studies reported declining self-assessed empathy at the clinical phase in both undergraduate education [16, 19–21] and during postgraduate residency [22, 34], unchanged stable empathy was found in our study, which was consistent with very few previous studies [35]. Some studies reported that the resident’s empathy score, measured using the same JSE, was comparable to our results [23, 36], and others reported increased results [34, 37]. As mentioned in an earlier study [38], the timing of clinical training varies by country, as does the number of years it takes to graduate. Therefore, differences in maturity may have led to differences in cognitive empathy by country.

On the other hand, we found decreased communication behavior in the emotional expression category for trainees, which may suggest that cognitive measures of empathy may not be completely in accordance with behavioral measures. Our finding was inconsistent with the results of an earlier study using the same measurements as ours, as this earlier study examined the relationship between communication behavior of medical students and their self-reported empathy and found that emotional responsiveness was among the predictors of the self-assessed empathy score [39].

One explanation for the decline in emotional expression in medical interviews could be that trainees are becoming more focused on their diagnosis and skills, which they view as crucial factors in treatment success. Our finding that trainees engaged in more data gathering, including a history of the current dental problem, would support this explanation. Holmes and colleagues [40] reported in their qualitative study exploring medical students’ clinical clerkship experience that students realized meeting a patient was a matter of gathering the information needed to make a diagnosis and present the information to the mentor.

Another possible explanation is that there was no change in empathy at the cognitive level because the measure used was a self-reporting instrument. It is possible that the trainees understand the need for empathy but find it difficult to express it in their behavior. Since the trainees are at the early stages of their careers, they may be unable to both collect relevant information for an accurate diagnosis and respond to patients’ emotions empathetically to draw out their concerns at the same time. It may take longer for them to learn to combine the ‘science’ of diagnosing and the ‘art’ of expressing empathy together in the medical interview. This speculation needs to be investigated in future research.

Another reason for the decline in the SP assessment regarding trainees’ understanding of SPs’ worries could be attributed to the decrease in the trainees’ empathic communication. When the trainees do not demonstrate much empathy in their communication with SPs, SPs may perceive that the trainees are reluctant to understand them. The decrease in SPs’ empathic expression may also be related to the decline of the SPs’ assessments, because communication is a reciprocal interaction. The decrease in the trainees’ legitimizing and empathic communication may have prevented patients from raising concerns. This was consistent with our previous study [41].

Moreover, some studies that showed an increase in dental students’ empathy noted this could be due to recently-completed communication lectures and practices [24]. Training in communication skills, including role playing with SPs who provide feedback, is effective in increasing empathy, but the effect is not sustained [25]. Although we provided medical interviewing training just before the start of this study, specific communication instruction focused on empathy was not implemented during the rest of their residency in the present study. Thus, it may be helpful to regularly provide some practice focused on empathetic communication skills during their training period. Although we have employed a portfolio for trainees to reflect on their practice, as well as for their instructors to review, the current system has not resulted in more empathetic students. Therefore, instructors may need to emphasize feedback not only on the trainees’ manual skills but also on patient communication.

This study had several limitations. First, it was conducted at a single institution with a small sample size. Second, we cannot eliminate the potential influence of gender on communication during the medical interview. Third, we also cannot exclude the potential observer bias which might affect SPs’ assessment. As the SPs were notified of the start and the end of the training, they may have expected the trainees to have improved drastically at the end of the training, which may have made their evaluations stricter than at the beginning. Fourth, we only analyzed communication during the medical interview and excluded other interactions, which may have affected the measurement of the behavioral aspect. In addition, we included nonverbal behaviors with utterances in the analysis, but did not include nonverbal behaviors without utterances in the analysis. Moreover, we only compared the number of communication behaviors and did not consider the context of where the communication behaviors were seen, which may limit results. It is desirable to include qualitative approach to fully understand empathy. It must also be noted that the trainees knew that the medical interviews were being video-taped and therefore it is possible that trainees may have been unable to act the way they usually do, or on the contrary, they may have behaved better than what they actually do in their daily practice. Lastly, the validity of the two cases should have been examined to further improve the validity of the study. Therefore, caution must be exercised before generalizing these findings. Further studies are required to verify these findings.

## Conclusions

A one-year postgraduate dental training program may be insufficient to foster empathy both cognitively and behaviorally among trainee dental students and fulfill SPs’ satisfaction during the medical interview. Providing communication-focused training and instructor feedback on trainees’ empathic communication with patients regularly during clinical training may enhance empathy. Nevertheless, further research is required to demonstrate the effectiveness of these educational methods with more certainty.

## Data Availability

The datasets used and/or analyzed during the current study are not publicly available since the postgraduate student dentists’ confidential information are included, but are available from the corresponding author on reasonable request.

## Abbreviations

SPs: simulated patients; JSE: Jefferson Scale of Empathy; RIAS: Roter Interaction Analysis System

## Authors’ information

Toshiko Yoshida, MA, PhD, is an assistant professor at Center for Education in Medicine and Health Sciences (Dental Education), Okayama University Graduate School of Medicine, Dentistry, and Pharmaceutical Sciences, Okayama, Japan.

Sho Watanabe, DDS, PhD, is a clinical fellow at Comprehensive Dental Clinic, Okayama University Hospital, Okayama, Japan.

Takayuki Kono, DDS, PhD, is an assistant professor at Comprehensive Dental Clinic, Okayama University Hospital, Okayama, Japan.

Hiroaki Taketa, DDS, PhD, is an assistant professor at Comprehensive Dental Clinic, Okayama University Hospital, Okayama, Japan.

Noriko Shiotsu, DDS, PhD, is a clinical fellow at Comprehensive Dental Clinic, Okayama University Hospital, Okayama, Japan.

Hajime Shirai, DDS, PhD, is a lecturer at Comprehensive Dental Clinic, Okayama University Hospital, Okayama, Japan.

Yukie Nakai, DDS, PhD, is a professor at Department of Dental Hygiene, University of Shizuoka, Junior College, Shizuoka, Japan.

Yasuhiro Torii, DDS, PhD, is a professor at Comprehensive Dental Clinic, Okayama University Hospital, Okayama, Japan.
